# IL-27 deficiency inhibits proliferation and invasion of trophoblasts via the SFRP2/Wnt/β-catenin pathway in fetal growth restriction

**DOI:** 10.7150/ijms.80684

**Published:** 2023-02-05

**Authors:** Xin-Yan Zhang, Xue-Yun Qin, Hui-Hui Shen, Ke-Tong Liu, Cheng-Jie Wang, Ting Peng, Jiang-Nan Wu, Shi-Min Zhao, Ming-Qing Li

**Affiliations:** 1 Laboratory for Reproductive Immunology, Hospital of Obstetrics and Gynecology, Fudan University, Shanghai 200080, People's Republic of China; 2 NHC Key Lab of Reproduction Regulation, Shanghai Institute for Biomedical and Pharmaceutical Technologies, Fudan University, Shanghai 201203, People's Republic of China; 3 Department of Obstetrics, Hospital of Obstetrics and Gynecology, Shanghai Medical School, Fudan University, Shanghai 200011, People's Republic of China; 4 Clinical Epidemiology, Hospital of Obstetrics and Gynecology, Shanghai Medical School, Fudan University, Shanghai 200011, People's Republic of China; 5 State Key Laboratory of Genetic Engineering, Collaborative Innovation Center for Genetics and Development, School of Life Sciences, Fudan University, Shanghai 200433, People's Republic of China; 6 Shanghai Key Laboratory of Female Reproductive Endocrine Related Diseases, Hospital of Obstetrics and Gynecology, Fudan University, Shanghai 200080, People's Republic of China

**Keywords:** Fetal growth restriction (FGR), IL-27, IL-27 receptor / IL-27RA, trophoblast cells, HTR-8/SVneo, canonical Wnt pathway, Wnt/β-catenin signaling pathway, SFRP2

## Abstract

**Background:** Fetal growth restriction (FGR) is characterized by restricted fetal growth and dysregulated placental development. The etiology and pathogenesis still remain elusive. IL-27 shows multiple roles in regulating various biological processes, however, how IL-27 involves in placentation in FGR pregnancy hasn't been demonstrated.

**Methods:** The levels of IL-27 and IL-27RA in FGR and normal placentae were determined by immunohistochemistry, western blot and RT-PCR. HTR-8/SVneo cells and *Il27ra^-/-^* murine models have been adopted to evaluate the effects of IL-27 on the bio-functions of trophoblast cells. GO enrichment and GSEA analysis were performed to explore the underlying mechanism.

**Findings:** IL-27 and IL-27RA was lowly expressed in FGR placentae and administration of IL-27 on HTR-8/SVneo could promote its proliferation, migration and invasion. Comparing with wildtypes, *Il27ra^-/-^* embryos were smaller and lighter, and the placentae from which were poorly developed. In mechanism, the molecules of canonical Wnt/β-catenin pathway (*CCND1*, *CMYC*, *SOX9*) were downregulated in *Il27ra^-/-^* placentae. In contrast, the expression of *SFRP2* (negative regulator of Wnt) was increased. Overexpression of *SFRP2 in vitro* could impair trophoblast migration and invasion capacity.

**Interpretation:** IL-27/IL-27RA negatively regulates SFRP2 to activate Wnt/β-catenin, and thus promotes migration and invasion of trophoblasts during pregnancy. However, IL-27 deficiency may contribute to the development of FGR by restricting the Wnt activity.

## Introduction

Fetal growth restriction (FGR), also known intrauterine growth restriction (IUGR), is one of the most common obstetric complications, affecting around 10% pregnancies worldwide. FGR is defined as fetal weight below the 10th percentile for gestational age and tightly related to adverse pregnancy outcomes and perinatal mortality 1. Causes for FGR include maternal malnutrition, metabolic or chronic wasting diseases, uteroplacental problems (such as multiple gestation, uterine malformations, and aberrant placentation), fetal chromosomal abnormalities, virus infection, and genetic alternations 2. Importantly, abnormal development of placenta is the predominant pathologic alteration as well as the leading cause that could be to blame for FGR 3, 4.

As the link between mother and fetus, placenta presents diverse functions including oxygen exchange, nourishment, waste removal, hormone production and immunity 5. The placenta is mainly composed of trophoblast cells, which are also of great significance to placental and fetal development 6, 7. During the implantation stage in normal pregnancy, the trophectoderm around the blastocyst invades into decidua and forms cytotrophoblasts (CTBs) which could further differentiate into outer layer multinucleated syncytiotrophoblasts (STBs) and thus forms the outer epithelial layer of the chorionic villi. Of note, differentiated trophoblasts involve in placentation via invading and remodeling the uterine vasculature during the first trimester. However, as for pathogenesis of FGR, trophoblast invasion is shallow and the utero-placental circulation is poorly built, which could limit material exchange and nutrient supply 8. To date, a series of dysregulated cytokines and signaling pathways have been reported in the development of FGR for their capacity in regulating trophoblast invasion 9.

IL-27, a heterodimeric cytokine consisted of the p28 subunit and the Epstein-Barr virus-induced gene 3 (EBI3) protein, is primarily generated by antigen presenting cells 10, and the intracellular signaling of IL-27 is initiated by the combination of IL-27 and IL-27 receptors (IL-27R) 11, 12. In detail, IL-27R includes two proteins: gp130 and IL-27α (IL-27RA) 13. Recently, accumulating studies have reported the role of IL-27 signaling in carcinogenesis, infectious diseases and autoimmune diseases for its capacity of regulating the innate or adaptive immune response and inducing immune tolerance 14-17. Our previous work has unraveled that IL-27 could stimulate IL-10 production and facilitate growth and invasion of endometrial stromal cells, contributing to the occurrence and progression of endometriosis 18, 19. We also found that IL-27 could activate decidual COX-2^+^ M2-like macrophage differentiation, indirectly facilitating decidualization, trophoblast invasion, and maternal-fetal tolerance 20, 21. However, whether IL-27 show effects on bio-functions of trophoblast cells and accounts for FGR development remain unexplained.

Herein, we identified the downregulation of IL-27/IL-27RA in FGR placentae in comparison to those of normal pregnancies, and demonstrates, for the first time to our knowledge, the regulatory role of IL-27 on proliferation, invasion and migration ability of trophoblast cells via Wnt/β-catenin pathway probably. Our results unveiled IL-27-mediated upstream regulatory pathway for trophoblast cells in normal pregnancy and aberrant changes in FGR, which helped clarify the placentation process and offered insights of potential therapeutic targets for FGR.

## Materials and methods

### Participants and placental samples

This study was approved by the Ethical Committee of the Obstetrics and Gynecology Hospital, Fudan University (#fckyy2022-113), and all recruited participants signed the informed consent. Patients were women of bearing age (20-45 years old), attending Obstetrics and Gynecology Hospital of Fudan University from February to October in 2022. FGR was defined as fetal birth weight <2500g and gestational age ≥37 weeks. Importantly, the gestational age has been calculated and corrected by ultrasound examination when the pregnancy was firstly diagnosed. Besides, the diagnosis of FGR was confirmed by experienced obstetricians when enrolled in. Exclusion criteria included those who had infectious diseases, other pregnancy-related diseases (gestational hypertension, preeclampsia, gestational diabetes mellitus,* etc.*), a history of malignancies, and previous adverse pregnancy outcomes. The placentae of FGR pregnancy and healthy term birth were collected immediately after the vaginal delivery. For each placenta, eight biopsies of the chorionic plate were taken from the fetal side (two from each of the four quadrants). Collected placental samples were immersed in sufficient Dulbecco's modified Eagle's medium (DMEM)/F-12 (Gibco) medium and carefully transferred on ice for further *in vitro* assays in laboratory.

### Animals

All animal procedures in this study were approved by the Department of Laboratory Animal Science, Fudan University (#2022JS-fckyy-059). *Il27ra^+/-^* mice (brought from Shanghai Model Organisms Center Inc., China) were raised in an SPF experimental animal facility, with temperature and humidity strictly controlled (22-25 ℃ 70% humidity). Female (8-week-old, around 18 grams) and male mice were caged at the ratio of 2:1 overnight, and the successful mating was confirmed by the presence of vaginal plug (P 0.5 day). At day 14.5 of pregnancy, female mice were sacrificed, and embryos and placentae of which were dissected along uterine longitudinal axis. Weight and the crown-rump were measured between the wildtype (*Il27ra^+/+^*) and knockout (*Il27ra^-/-^*) embryos, meanwhile the placental weight and diameter were also recorded. Of note, only the same litter that contained both wildtype and knockout embryos was included in statistical analysis, otherwise, they would be excluded.

### Cell cultivation and treatment

The HTR-8/SVneo trophoblast cell line was purchased from the Chinese Academy of Sciences Cell Bank (Shanghai, China) and cultured in Dulbecco's modified Eagle's medium (DMEM)/F-12 (Gibco), supplemented with 10% fetal bovine serum (Gibco, 10270-106) and 1% penicillin-streptomycin Solution (100×, Beyotime, C0222). The cultivation condition was set at 37°C, with a humidified atmosphere containing 5% CO_2_ in the incubator (Heal Force). HTR-8 trophoblast cells were seeded in 6-well or 96-well Costar plates (Corning), and were stimulated with different concentrations of IL-27 (Peprotech, New Jersey, USA) after adhesion for 24 or 48 h.

### Cell transfection

SFRP2 overexpression (SFRP2-OE) plasmid and control plasmid were from Beijing Tsingke Biotechnology Co., Ltd. (China). HTR-8 were seeded in 6-well culture plates and subsequently infected with SFRP2-OE or control plasmid packaged in lentivirus at the exponential phase for 48 h. Next, fresh complete medium was substituted for further cultivation. The infection efficiency was evaluated by RT-PCR and western blot 48 h after infection.

### Immunohistochemistry and Immunofluorescence

Paraffin sections (4µm) of the placental tissues derived from FGR or normal pregnancy were dewaxed in xylene and rehydrated in graded ethanol. Antigens retrieval was performed by microwave method in citrate buffer (0.01M, pH = 6.0), and Triton X-100 (0.5%, diluted with 1×PBS) was used for permeabilization. 3% hydrogen peroxide and 10% goat serum were applied to quench endogenous peroxidase activity and non-specific binding, respectively. Then the slides were incubated with specific murine monoclonal antibodies against IL-27 (Novus Biotechne, Colorado, USA, NBP2-16951, 1:100) or IL-27RA (Santa Cruz Biotechnology, Dallas, USA, sc-376309, 1:100) overnight in a humid chamber, meanwhile added only 1×PBS to controls. After washing three times with PBS, the sections were overlaid with peroxidase-conjugated goat anti-mouse/rabbit IgG and the reaction was developed with GTVisionTM Ⅲ Detection System (Gene Tech, Inc, Shanghai, China) following the manufacturer's instructions. Thereafter, counterstaining was performed using the hematoxylin staining solution.

Immunofluorescence staining was performed as our previous protocol to evaluate the trophoblast invasion in murine placentae and the accumulation of β-catenin in HTR-8, respectively 21, 22. Murine placentae were labeled with rabbit anti-mouse cytokeratin 7 (CK7) antibody (Abcam, ab9021,10 μg/ml) and the HTR-8 was labelled with β-catenin (Cell Signaling Technology, Massachusetts, USA, 8480, 1:200). Nuclei were stained with 4ʹ,6-diamidino-2-phenylindole (DAPI; Beyotime, C1006).

### Western blotting

Cells were treated with RIPA Lysis Buffer (Beyotime, P0013B) containing 1% phenylmethanesulfonylfluoride (PMSF) for protein extraction. While for placental tissues, the lysis process was assisted with tissue homogenizers (Miltenyi). BCA protein assay kit (Beyotime, P0012) was applied to quantify protein concentration. The lysates were then boiled (10 min, 95°C) for degeneration. Protein (25 µg) was loaded on 10% SDS-PAGE gel with markers and electrophoresed by using a Miniprotein III system (Bio-Rad, 1658033). Afterward, it was transferred to PVDF membranes (Millipore, ISEQ00010) for 90 min and immersed in 5% skimmed milk for 1 h at room temperature for blocking non-specific binding. The PVDF membranes were incubated with primary antibodies against IL-27 (Novus Biotechne, Colorado, USA, NBP2-16951, 1:500), IL-27RA (Santa Cruz Biotechnology, Dallas, USA, sc-376309, 1:1000), MMP2 (Cell Signaling Technology, Massachusetts, USA, 40994, 1:500), MMP9 (Cell Signaling Technology, Massachusetts, USA, 13667, 1:500), SFRP2 (Santa Cruz Biotechnology, Dallas, USA, sc-365524, 1:500), WNT3A(Santa Cruz Biotechnology, Dallas, USA, sc-74537, 1:500) or β-catenin (Cell Signaling Technology, Massachusetts, USA, 8480, 1:500) overnight at 4°C respectively. Then PVDF membranes were washed with PBST solution for three times and treated with peroxidase-conjugated goat anti-rabbit IgG secondary antibody (1:5000; Bioworld Technology, BS10003) or goat anti-mouse IgG secondary antibody (1:5000; Bioworld Technology, BS10003) at room temperature for 1h. After adequate washing, Immobilon Western Chemiluminescent HRP Substrate Kit (Millipore, WBKLS0100) was used for chemiluminescence detection and Image J software was applied for relative quantification.

### Real time (RT)-PCR and genotyping

Total RNA was extracted from human or murine placental tissues or cells by using Fast Tissue RNA Purification Kit (EZBioscience, EZB-RN5), which was then reverse-transcribed into cDNA with 4×Reverse Transcription Master Mix (EZBioscience, A0010GQ). Quantitative reverse transcription-PCR (RT-qPCR) was performed using 2× Color SYBR Green qPCR Master Mix (ROX2 plus) (EZBioscience, A0012-R2) and the analysis was based on ABI Prism 7900 Fast Sequence Detection system (Thermo Fisher Scientific). The fold change at the transcriptional level of the target genes was displayed by calculating the 2^-ΔΔCt^ method and each sample was run in triplicates. Relative mRNA expression levels were normalized to β-actin (ACTB**)**. Sequences of each pair of primers was displayed in **Table 1**.

Moreover, EZ-press Tissue Direct PCR Kit (EZBioscience, EZB-TDP1) was used to genotype *Il-27ra* allele of dissected murine embryos as manufacturer's instructions. PCR products were then amplified with the following primers: Wild-type Forward: 5′-CGCAGAAAGTTCTCATCT-3′; Wild-type Reverse: 5′- TCATACAGTACCCATCCC-3′; Knockout Forward: 5′- ACCGTAAAGCACGAGGAA-3′. Knockout Reverse: 5′- GACCACCAAGCGAAACAT -3′. Next, 2% agarose gel in TBE buffer (90 mM Tris, 90 mM boric acid, 2 mM EDTA) was prepared for electrophoresis at 90 V for 60 min.

### Cell Counting Kit-8 (CCK-8) proliferation assay

HTR-8/SVneo cells were plated in 96-well plates (2,000 cells per well), treating with or without IL-27 (0, 1, 10 and 100 ng/ml) for 24 or 48 h. And there were six replicates for each sample at each concentration. The proliferation of the HTR-8/SVneo cells was determined with a Cell Counting Kit-8 (Beyotime, C0038). Before adding CCK-8, the medium was removed and adherent cells was washed with PBS twice. Next, 10 µl CCK-8 and 100 µl fresh complete medium was added into each well (with or without cells) and incubated at 37°C for 2 h in the dark. Finally, the optical density (OD) at the wavelength of 450 nm was measured by a microplate spectrometer (BioTek Instruments, Inc., Winooski, VT, USA). The proliferation index was calculated as follows:

(A_450_(Treated group)-A_450_(Blank)) / (A_450_(Control group)-A_450_(Blank))

Treat group: HTR-8 treated with 1, 10 or 100 ng/ml IL-27

Control group: HTR-8 treated with 0 ng/ml IL-27

Blank: Blank well without cells but added CCK-8

### Migration and Matrigel invasion assays

Transwell migration/invasion assays were performed as our previous procedure 23. In brief, 2 × 10^4^ HTR‐8/SVneo cells and 200 μl serum-free DMEM/F12 were pipetted to mix thoroughly, suspension of which was added to the upper compartment of Corning Costar^®^ transwell inserts (8 μm-pore size, 6.5 mm diameter) pretreated with Matrigel (60 μL of 1 mg/mL for invasion assays) or not (for migration assays). For the lower chamber, 600 μl complete medium was added. To evaluate the potency of IL-27 in regulating the invasiveness of HTR-8, the optimal concentration of IL-27 was added or not both in the upper and lower chamber. The transwell system was then cultured at 37 °C, 5% carbon dioxide incubator for 48 h. Afterward, the transwell was taken from the 24-well plate and gently washed three times with PBS, penetrated in 4% paraformaldehyde for 30 min and stained with 0.1% crystal violet for 20 min. Pictures were obtained under an inverted microscope (Olympus) at five random visual fields for each section. Relative invasion ability was evaluated by the ratio of the number of invaded cells in IL-27 treated group to those in controls. Each sample was carried out in triplicates, and repeated three times independently.

Additionally, cell migration was measured by the scratch assay. In 6-well plates, a 1000 µl sterile pipette tip was used to streaked a line at the bottom of each well, which was then allowed to recover in untreated or IL-27(100 ng/ml)-treated complete medium for 24 h in the incubator. The plates were photographed at 4× and width of scratched wound was measured at 10× magnification (Olympus) at 0 h and 24 h, respectively. Migration was calculated by the ratio of the width at 0 h to that at 24 h.

### RNA-seq Analysis

Wildtype (n = 3) and knockout (n = 3) murine placentae from different litters were sent for RNA-seq analysis performed by Novel Bioinformatics Co. Ltd. (Shanghai, China), and one sample was discarded due to sequencing quality issues. Further analysis was supported by R software (version 4.1.0). DESeq2 package was utilized to evaluate differential gene expression. Genes of which absolute fold-change over 1.5 and FDR less than 0.05 were considered significantly different. The significance of enrichment for the differentially expressed genes was determined by using the hypergeometric test, and p-value less than 1×10^-5^ were regarded significantly enriched. Finally, enriched genes were annotated with GO terms obtained from the Gene Ontology database (http://www.geneontology.org/). Annotated genes were considered to enrich in certain pathways if the p-value was less than 0.05. Additionally, gene set enrichment analysis (GSEA) was performed using GSEA software (http://software.broadinstitute.org/gsea/).

### Statistical analysis

All statistical analyses and graphs were conducted with the software GraphPad Prism (version 9.0, CA, USA), and all data were presented as the mean ± standard deviation. Unpaired Student's t-test was used for comparison between two groups. For more than two subgroups, one-way ANOVA was applied, and p-value was adjusted by Bonferroni correction for multiple comparison. Statistical significance is indicated mainly by one asterisk (*) for P < 0.05, two asterisks (**) for P < 0.01, three asterisks (***) for p<0.001, and four asterisks (****) for p<0.0001.

## Results

### IL-27 and its receptor IL-27RA are reduced in FGR placentae

**Figure 1:**
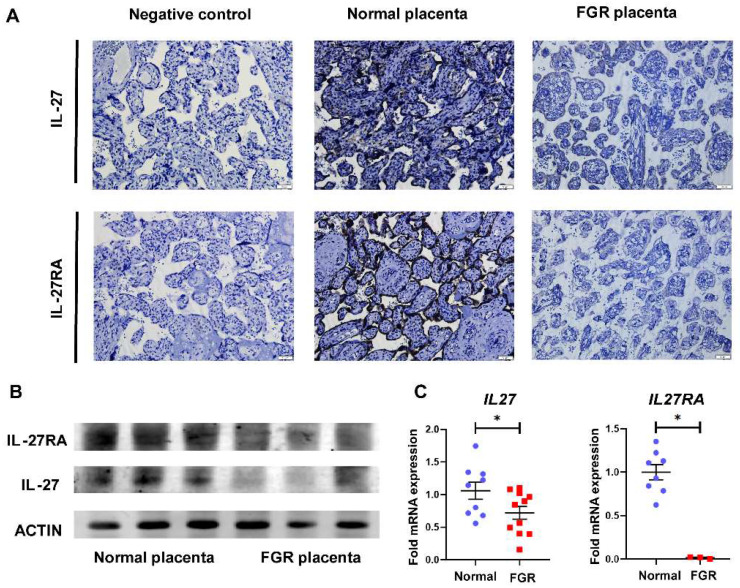
Expression of IL-27 and IL-27RA in human normal and FGR placentae. (A) IHC staining of IL-27 and IL-27RA in villus structures derived from negative control (sections of normal placenta only treated with secondary antibodies), normal placenta (n = 5) and FGR placenta (n = 5). (B) Western blotting of IL-27 and IL-27RA in normal or FGR placenta respectively. (C) Relative expression of *IL27* (normal (n = 9) *vs.* FGR (n = 11)) and IL*27RA* (normal (n = 8) *vs.* FGR (n = 3)) performed by tissue RT-PCR between FGR and normal placenta. Statistical analyses were performed with unpaired t test. *P < 0.05.

To explore the role of IL-27 and its receptors in FGR pregnancies, we first perform IHC staining on normal and FGR placentae. Considering GP130, another subunit of IL-27 receptor, is also shared with IL-6 24, thus we focused on IL-27RA to figure out the specific effect IL-27 takes in FGR and normal pregnancy. As displayed in **Figure 1A**, staining of both IL-27 and IL-27RA were obviously decreased in the villi of FGR placenta in comparison to normal controls. Similar results were also observed in Western-blot and RT-PCR, which demonstrated downregulated IL-27 and IL-27RA in FGR placentae at protein or mRNA level, respectively (**Figure 1B, C**).

### IL-27 promotes proliferation, migration and invasion of trophoblasts* in vitro*

**Figure 2:**
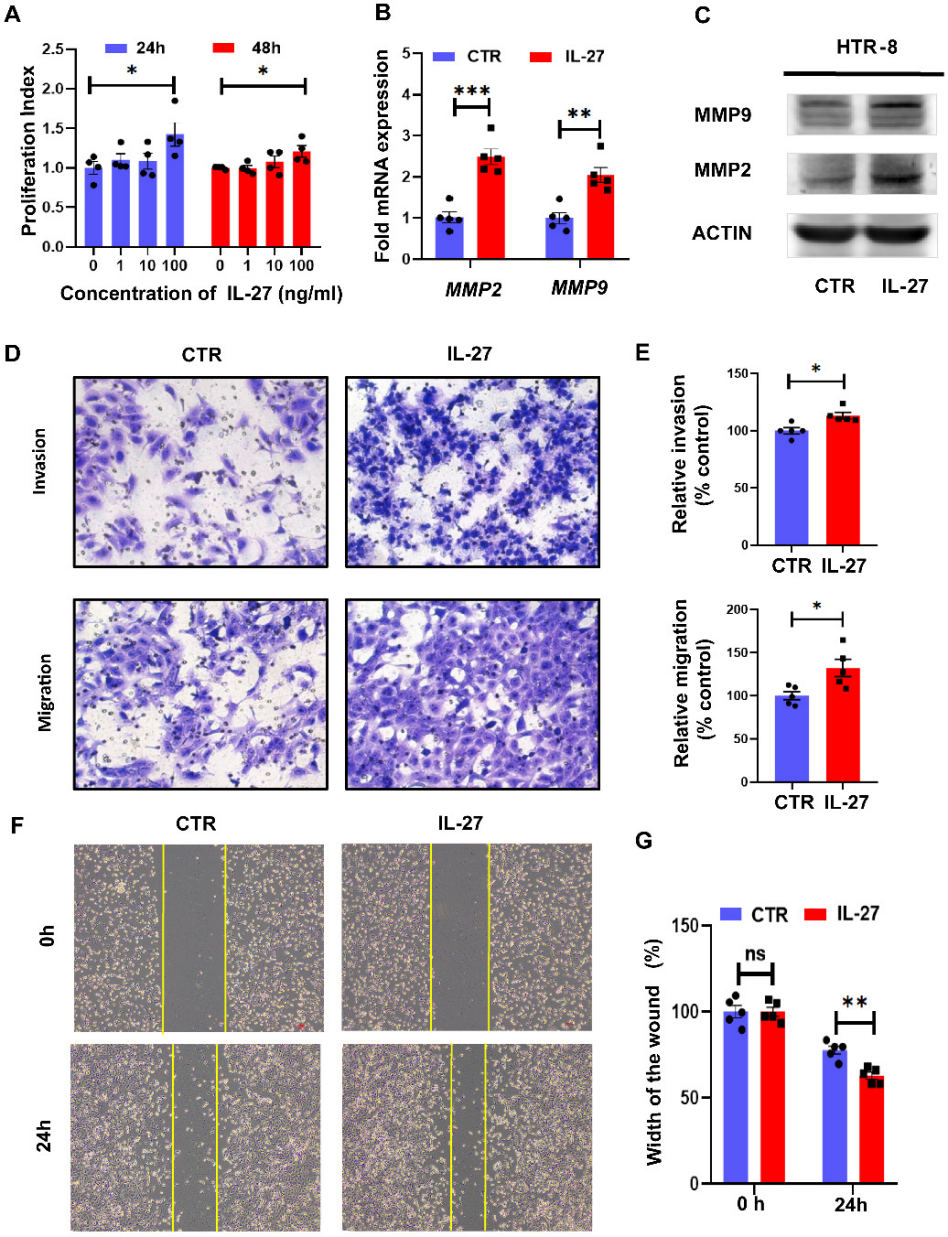
IL-27 treatment promotes proliferation, migration and invasion of trophoblast cells *in vitro.* (A) Different concentrations of IL-27 (0, 1, 10, 100 ng/ml) were treated on HTR-8/SVneo for 24h or 48h. (B, C) MMP2 and MMP9 expression levels in IL-27 treated (100 ng/ml, 24h) HTR-8 or the controls were evaluated by qPCR and western blotting. (D, E) Transwell systems (Corning Costar® transwell inserts, 8 μm-pore size, 6.5 mm diameter) with Matrigel (60 μL of 1 mg/mL for invasion assays) or not (for migration assays) was applied to compare invasion and migration ability between IL-27 treated HTR-8 (n = 5) and controls (n = 5). Relative quantification was analyzed by Image J on the left. (F, G) Scratch test: IL-27 treatment (100 ng/ml) or not for 24h or 0 h on HTR-8 (n = 5), and the width of scratched wound was recorded. CTR, control; MMP-2/9: Matrix metallopeptidase 2/9. Statistical analyses were performed with unpaired t test. *P < 0.05; **P < 0.01; ns, not significant.

Trophoblast cells are vital for the establishment of maternal-fetal interface, and their proliferation, invasion and migration abilities are recognized to determine the placental attachment and growth in normal pregnancy. FGR, typified by restricted fetal growth and maldeveloped placenta, was tightly connected to dysregulated bio-functions of trophoblast. Therefore, we next investigated the role of IL-27 in regulating bio-functions of trophoblast cells based on HTR-8//SVneo cell line. As for different concentrations (0, 1, 10, 100 ng/ml) of IL-27 treating for 24 h or 48 h, HTR-8 treated with 100 ng/ml IL-27 achieved the higher proliferation index, while the proliferation index was comparable between 24h treatment with 100 ng/ml IL-27 and that for 48h (**Figure 2A**). Hence, 100 ng/ml IL-27 treating for 24h was recognized as the optimal condition for promoting cell proliferation in HTR-8. Moreover, MMP9 and MMP2, molecular markers for invasiveness, showed distinct upregulation at both mRNA and protein levels in IL-27 treating group when comparing to the control (**Figure 2B, C**). According to transwell systems, both invasion and migration could be promoted by IL-27 (100 ng/ml), which was also confirmed by the relative quantitative analysis (**Figure 2D, E**). Besides, as an effective strategy to evaluate trophoblastic migration, the scratch test was also performed. IL-27 treatment did facilitate the recovery of the wound (**Figure 2F, G**). To summarize, IL-27 positively regulates trophoblastic bio-functions, including cell proliferation, migration and invasion abilities.

### IL-27RA deficiency inhibits embryo growth and placenta development* in vivo*

**Figure 3:**
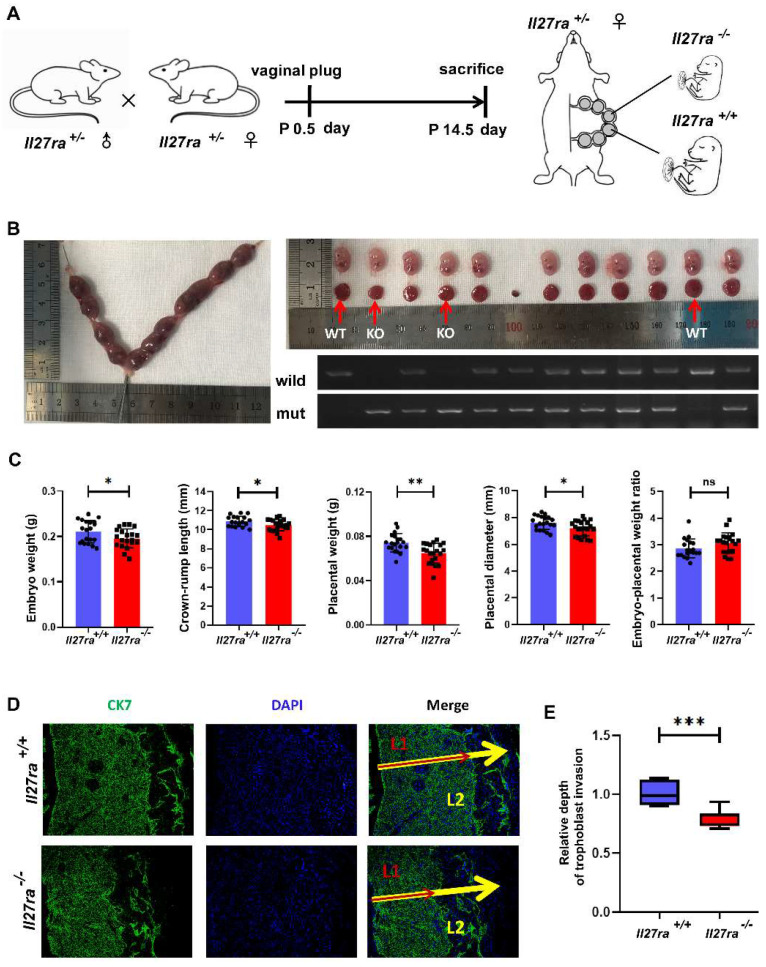
*Il27ra^-/-^* embryos present FGR phenotypes. (A) *Il27ra^+/-^* mice were caged together overnight, and the occurrence of vaginal plug was regarded as 0.5 day of pregnancy. At P14.5, pregnant mice (n = 13) were sacrificed. (B) Embryos and placentae of littermates were separated and the genotypes of these embryos were identified. (C) Embryo weight, Crown-rump length, placenta weight, placenta diameter and embryo-placental weight ratio were recorded accordingly, and the differences were analyzed between *Il27ra^+/+^* (n = 22) and *Il27ra^-/-^*(n = 24). (D, E) Penetrate depth of CK7^+^ trophoblasts (L1: combination of labyrinth and junctional zone layers) in placentae (L2: full-thickness of the placenta) of WT (n = 7) and KO (n = 7) were compared by immunofluorescence. The relative depth was calculated as the ratio of L1/L2. WT, wildtype: *Il27ra^+/+^*, KO, knockout: *Il27ra^-/-^*. Statistical analyses were performed with unpaired t test. *P < 0.05; **P < 0.01; ***P < 0.001.

To further investigate the effect of IL-27 deficiency on placenta and fetal growth, *Il27ra*-knockout heterozygous mice (*Il27ra*^+/-^) were crossed and the pregnant mice were sacrificed at 14.5 day of pregnancy (**Figure 3A**). Afterwards, embryos and placentae were separated respectively, and the embryos were analyzed for genotyping (**Figure 3B**). In a stark contrast to wildtype (*Il27ra*^+/+^) ones, *Il27ra*-knockout (*Il27ra*^-/-^) embryos were lighter with reduced crown-rump length, and the placentae of which presented lower weight and smaller diameter. However, there showed no significant difference of embryo-placental weight ratio between groups (**Figure 3C**). As a widely acknowledged marker for trophoblast cells, CK7 was adopted to present the infiltration of CK7 ^+^trophoblast cells into murine uterus by immunofluorescence staining. It was displayed that poor trophoblasts invasion of murine placentae with decreased penetrated depth in *Il27ra*-knockout (*Il27ra*^-/-^) embryo group (**Figure 3D, E**).

### IL-27/IL-27RA exerts its effects in FGR pathogenesis via SFRP2/Wnt/β-catenin signaling pathway

**Figure 4:**
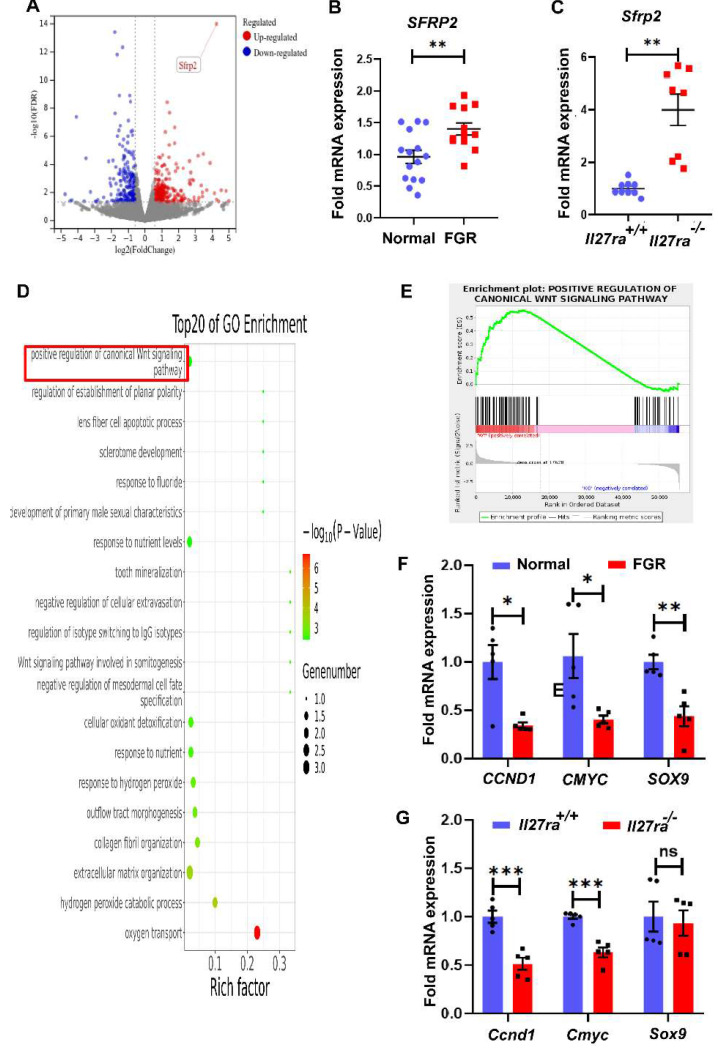
SFRP2 targeted Wnt/β‐catenin pathway is inhibited in *Il27ra^-/-^* embryos. Placentae derived from KO and WT embryos were sent for RNA-seq. (A) Volcano plot for DEGs in *Il27ra^-/-^* placentae. Genes were filtrated and marked for Fold change >1.5 and FDR < 0.05. Among upregulated DGEs, *Sfrp2* was overt as the least FDR and high fold change. (B, C) Expression of placental *SFRP2* was measured by tissue RT-PCR on human (normal (n = 15) *vs.* FGR (n = 11)) and murine model (WT (n = 9) *vs.* KO (n = 8)). (D) TOP 20 pathways of GO enrichment. (E) GSEA analysis for enriched canonical Wnt signaling pathway. (F, G) Downstream molecules of Wnt/β-catenin (*CCND1*, *CMYC*, *SOX9*) were measured by tissue RT-PCR on human (normal (n = 5, ≥37 gestational weeks) vs. FGR (n = 5, ≥37 gestational weeks)) and murine placentae (WT (n = 5) *vs.* KO (n = 5)). WT: *Il27ra^+/+^*, KO: *Il27ra^-/-^*. Statistical analyses were performed with unpaired t test. *P < 0.05; **P < 0.01; ***P < 0.001; ns, not significant.

To clarify the underlying mechanism of how the dysregulated IL-27/IL-27RA participates in FGR development, RNA-seq analysis was performed and a total of 66 differentially expressed genes (DEGs, ≥ 1.5-fold change in expression with ≤ 0.05 false discovery rate) were identified by placentae derived from wildtype and *Il27ra*-knockout mice. Among all DEGs, 24 genes were upregulated while 42 genes were downregulated. In the volcano plot, we noticed that secreted frizzled-related protein 2 (*Sfrp2*) presented prominent upregulation (**Figure 4A**), and subsequent RT-PCR did confirm the increase of *SFRP2* in FGR placentae comparing with the normal controls both for human and mice (**Figure 4B, C**).

We next grouped DEGs based on their individual signaling pathways through gene ontology (GO) enrichment analysis. Interestingly, as the pathway which SFRP2 negatively regulates, Wnt/β-catenin signaling was one of the top 20 pathways in GO enrichment (**Figure 4D**). Actually, Wnt/β-catenin signaling pathway has been well-recognized to played vital roles in malignancy processes via regulating cell proliferation, migration and invasion. To this end, we selected this pathway as the potential candidate for the following investigation. Gene Set Enrichment Analysis (GSEA) showed that enriched pathways related to Wnt/β-catenin pathways were highly activated in the wildtype group at the default cut-off FDR < 0.25 (Enrichment Score = 0.5553633, Normalized Enrichment Score = 1.3369913, FDR q-value = 0.03397341) (**Figure 4E**). In addition to the increased expression of *SFRP2*, other molecules that positively associated with Wnt/β-catenin activity, including *CCND1* and* CMYC*, were downregulated in both human and murine FGR placentae, while *SOX9* only achieved statistical significance in human FGR placentae (**Figure 4F, G**). Anyway, these results suggest that inhibited Wnt/β-catenin signaling induced by IL-27/IL-27RA deficiency may be correlated with FGR development.

### SFRP2 inhibits trophoblastic invasion via downregulating Wnt/β-catenin signaling

**Figure 5:**
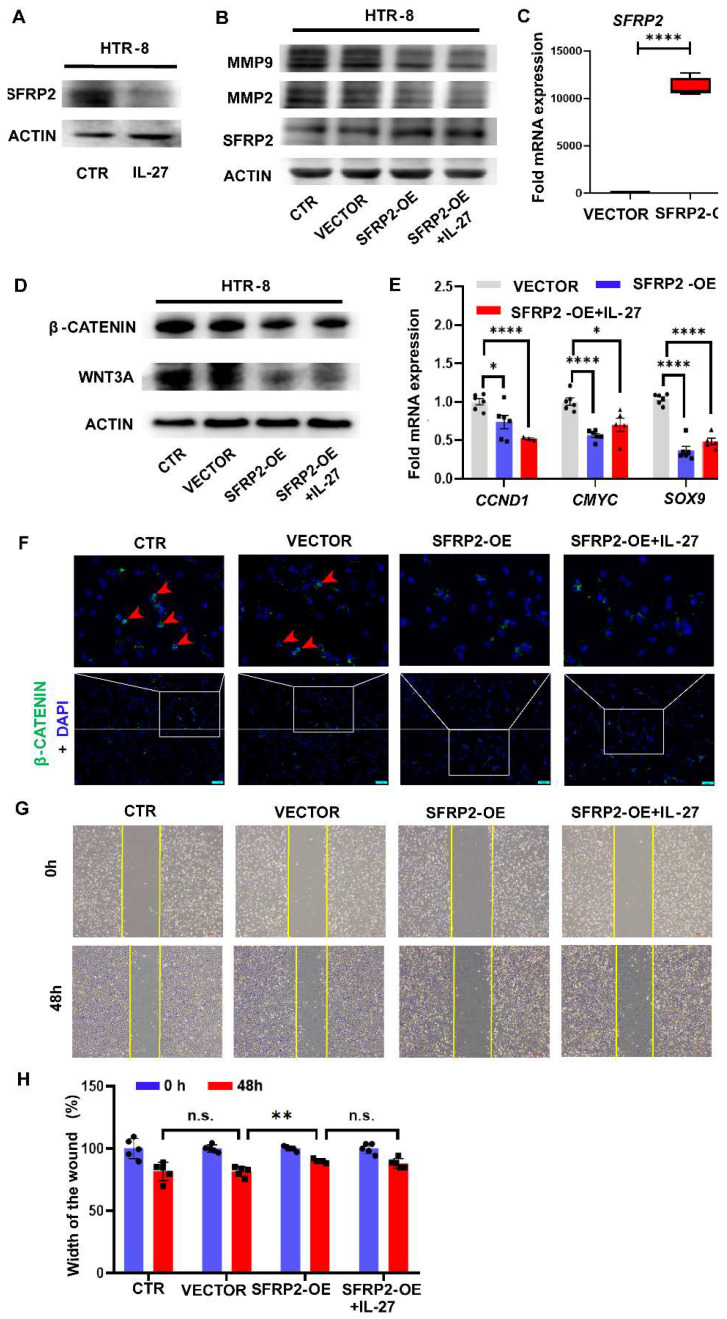
Overexpression of SFRP2 inhibits trophoblast migration and invasion via modulating canonical Wnt pathway. (A) Expression of SFRP2 with IL-27 treatment (100 ng/ml, 24 h) in HTR-8. Among groups of control, vector, SFRP2-OE and SFRP2-OE+IL-27: (B) Western blot was performed to evaluate expression of MMP2, MMP9 and SFRP2. (C) Expression of *SFRP2* was measured by RT-PCR to confirm the success of transfection. (D, E) Expression of WNT3A, β-catenin and downstream molecules of canonical Wnt pathway (*CCND1*, *CMYC*, *SOX9*) was measured by western blot and RT-PCR respectively. (F) Immunofluorescence staining of intracellular β-catenin. (G, H) Scratch test of SFRP2-OE HTR-8 with or without IL-27 supplementation. SFRP2-OE: SFRP2-overexpressed. Statistical analyses were performed with unpaired t test and one-way ANOVA (adjusted by Bonferroni correction for multiple comparison). *P < 0.05; **P < 0.01; ****P < 0.0001; ns, not significant.

To evaluate the role of SFRP2 in the Wnt/β-catenin pathway and regulating invasion ability of trophoblast cells mediated by IL-27 *in vitro*, we firstly detected the expression of SFRP2 in HTR-8 with IL-27 treatment and observed the inhibitory effect of IL-27 on SFRP2 expression at the protein level (**Figure 5A**). Next, we constructed SFRP2 overexpression (SFRP2-OE) plasmids and over-expressed the expression of SFRP2 in HTR-8 by transfection. The efficacy of transfection was verified by RT-PCR and western blot (**Figure 5B, C**). Moreover, SFRP2-OE strikingly decreased the expression of WNT3A, β-catenin and Wnt target genes (*CCND1*, *CMYC*, *SOX9*) when comparing to the controls (**Figure 5D, E**). Immunofluorescence staining also revealed reduction of accumulated intracellular β-catenin in SFRP2-OE trophoblasts (**Figure 5F**). These findings suggest SFRP2 as a suppressor of canonical Wnt pathway in HTR-8. Functionally, SFRP2-OE was able to attenuate the potency for HTR-8 to recover from the scratch wound (**Figure 5G, H**). Likewise, both MMP2 and MMP9, markers for invasion, were downregulated by exaggerated expression of SFRP2 (**Figure 5B**). However, both of these effects couldn't be abrogated by supplementing IL-27.

## Discussion

Chronologically, FGR is usually clinically recognized in the second or third trimester, while the potential pathophysiologic alterations might initiate earlier in the early pregnancy 25. FGR placentae show reduced volume in the first trimester with poor thickness and diameter. Additionally, differentiation of villus was inhibited in FGR placentae with reduced villous branching and decreased density of capillary in terminal villi 4. Trophoblast cells derived from FGR placentae also presented reduction in its proliferation, viability and invasion ability 26. According to our results, Il-27 and its specific receptor IL-27RA were obviously reduced in FGR placentae, and administration of exogenous IL-27 could elevate trophoblast proliferation, migration and invasion *in vitro*. However, Ge *et al.* have reported suppressive effects of IL-27 on invasion and migration of HTR-8/SVneo cells by regulating the epithelial-mesenchymal transition process through an STAT1 pathway 27. The different concentrations of IL-27 might be a decisive factor. They treated HTR-8/SVneo cells with 50 ng/ml IL-27, which didn't influence significant trophoblastic proliferation. While we designed a concentration gradient of IL-27 (0, 1, 10, 100 ng/ml), only 100 ng/ml was effective for HTR-8 to proliferate and we adopt 100 ng/ml as the optimal concentration of IL-27 for HTR-8 cells. From this prospective, it could be rational for them to observe inhibitory effect of IL-27 for migration and invasion of HTR-8 cells. Anyway, these findings implied significance of IL-27/IL-27RA signaling in FGR and revealed the regulatory role of IL-27 for bio-functions of trophoblast cells. Furthermore, the weight and size of embryos or placentae in the *Il27ra^-/-^* group were inferior to those of the wildtype. Depth of trophoblastic invasion in *Il27ra^-/-^* murine placentae was shallower than those of *Il27ra^+/+^* ones. Hence, the depletion of *Il27ra* could impede placental development and induce FGR-like phenotypes in mice.

According to RNA-seq analysis based on placentae from *Il27ra^-/-^* and wildtypes embryos, we noticed that one of the significantly upregulated DEGs with relatively high fold change was *Sfrp2*, and the upregulation of SFRP2 was confirmed in human FGR and *Il27ra*^-/-^ murine placentae respectively. Wnt/β‐catenin signaling (canonical pathway), is a vital and highly-conserved pathway for embryo development and involves in carcinogenesis for its role in regulating cell differentiation, proliferation and migration 28. The activation is elicited by the combination of Wnt protein and its membrane receptor frizzled protein, leading to upregulated free β-catenin in the cytoplasm, which is then translocated into the nucleus to function as a transcriptional coactivator of transcription factors that belong to the TCF/LEF family. In this way, target genes were regulated at transcriptional level. However, in inactivated status, β-catenin is assembled with a destruction complex that normally degrade it 28, 29. SFRP2 contains a cysteine-rich domain homologous to the Wnt-binding site of Frizzled proteins and act as soluble modulators of Wnt signaling 30. It used to be considered as an inhibitor for canonical Wnt signaling 31, 32. To date, dysregulated SFRP2 has been reported to involve in a series of physiological or pathological processes, for instance, cellular differentiation, tumor metastasis, drug resistance and functional alterations of stem cells 33-35. In consistence with highly-expressed *SFRP2* in FGR placentae, we also observed inactive Wnt signaling as downregulated Wnt target genes, including *CCND1*, *CMYC* and *SOX9* (genes regulate carcinogenesis, metastasis and drug resistance 36-39), though *Sox9* didn't present statistically significance in murine models. In addition to the sample size, different species and differentially expressed Wnt proteins may account for this discrepancy. Importantly, we found IL-27 presented an inhibitory effect on the expression of SFRP2 *in vitro*. SFRP2-overexpressed HTR-8 cells did inhibit the activation of Wnt signaling and presented impaired potential of migration and invasion. However, these effects couldn't be reversed by IL-27 supplement. In addition to relatively low expression of IL-27RA in HTR-8, the exact pattern by which IL-27 may regulate SFRP2/Wnt/β-catenin and other factors or signaling pathways which might involve in this process should be further studied. Anyway, these results suggested excessive *SFRP2* in FGR placentae may inhibit Wnt/β-catenin activity to turn off the transcription of downstream genes that were related to proliferation, invasion and migration of HTR-8 cells.

However, there have been some contradictory results about the exact roles of SFRP2 in regulating Wnt/β-catenin pathway 40, 41. For example, according to the experimental data from Sun *et al*., SFRP2 increased the oncogenic activities of WNT16B via facilitating cancer cell proliferation, migration, invasion and drug resistance 41. Actually, positive effect of SFRPs on Wnt signaling have been reported, especially that SFRP2 enhanced WNT3A-dependent phosphorylation of LRP6 and elicited β-catenin cytoplasmic accumulation and translocation nuclear thereafter 42, 43. One possible explanation is that SFRP2 function might exert in a tissue/cell-specific way. Marschall *et al.* reported enhancing effects of SFRP2 on Wnt3a signaling in HEK293A cells but there was no effect on murine fibroblasts^42^. However, we observed remarkable inhibitory roles of SFRP2 for WNT3A activity in HTR-8 cells, which is in line with the finding of Sorina *et al* 44. Actually, as a soluble factor, SFRP2 are proposed to act not only by directly binding to Wnt proteins but also directly with Frizzled proteins 45. Thus, different combination dependent on molecular and cellular context might lead to very different stories. It's still interesting to explore whether and how the exogenous supplementation of SFRP2 regulates Wnt signaling, by which bio-functions of trophoblast cells are modulated.

Some studies have also mentioned the potential relationship between IL-27 and Wnt/β-catenin pathway. Ruiz-Riol *et al.* reported that elevated levels of IL-27 are associated with higher viral load and increased size of the viral reservoir, which may be mediated by a dysregulated Wnt/β-catenin signaling pathway 46. Besides, Wnt/β-catenin pathway was essential for normal T-cell development and its deficiency eliminated the anti-inflammatory effect mediated by IL-27 47. STAT3, which is a downstream factor of IL-27/IL-27RA responsible for translocating into the nucleus and enhancing transcription 48, may interacts with Wnt/β-catenin through WNT5A 49. How IL-27 interacts with Wnt, especially WNT3A, deserves further investigation. Qiang et al. have demonstrated that Wnt-mediated migration might be mediated by protein kinase C (PKC) family (including PKCα, PKCβ, and PKCμ) which is vital for cytoskeletal changes and cell motility 50. Functionally, Wnt-mediated cell invasion may account for Wnt target genes such as CCND1 and matrix metalloproteinases (MMPs) 51. Interestingly, we found that IL-27 repressed the expression of SFRP2 *in vitro* and Wnt/β-catenin was inhibited in murine placentae derived from *Il27ra^-/-^* embryos. Bio-functions of proliferation, migration and invasion of HTR-8 were all enhanced with elevated expression of MMP2 and MMP9 after IL-27 stimulation *in vitro*, which indicating that the Wnt pathway may be involved in the regulation of trophoblast behaviors mediated by IL-27.

IL-27 is produced by trophoblasts at maternal-fetal interface at various stages of gestation 52. Actually, during placental development, on top of well-functioned trophoblast cells, there are other hallmark events that IL-27 may involve in: 1) decidualization of endometrium stromal cells; 2) infiltration of immune cells during decidualization and establishment of maternal-fetal immune tolerance 53. Whether IL-27 deficiency could disrupt the decidua's role as a nutrient sensor needs further investigation.

Moreover, IL-27 has multiple immunomodulatory effects on regulating innate immunity and adaptive immunity through interacting with various cells 21, 54-57. It has been reported to exert either pro- or anti-inflammatory effects on tumor microenvironment 39. Interestingly, skewed balance between different subtypes of immune cells has become a promising perspective to explore the pathogenesis of FGR 58, 59. Kristin *et al.* have identified defective progesterone-responsiveness of CD11c^+^ dendritic cells was associated with FGR placentation of mice 58. Romy* et al.* reported that FGR placentae displayed excessive infiltrated Treg cells and altered proportions to different subtypes of macrophages 59. Considering the ability of IL-27 to modulate immune response mentioned above, it will be worthwhile to explore whether IL-27 is involved in FGR placentation via influencing immune microenvironment at maternal-fetal interface. Altogether, negative effects of IL-27 shortage for trophoblast bio-functions could result from comprehensive microenvironment at maternal-fetal interface in FGR pregnancy.

**Figure 6:**
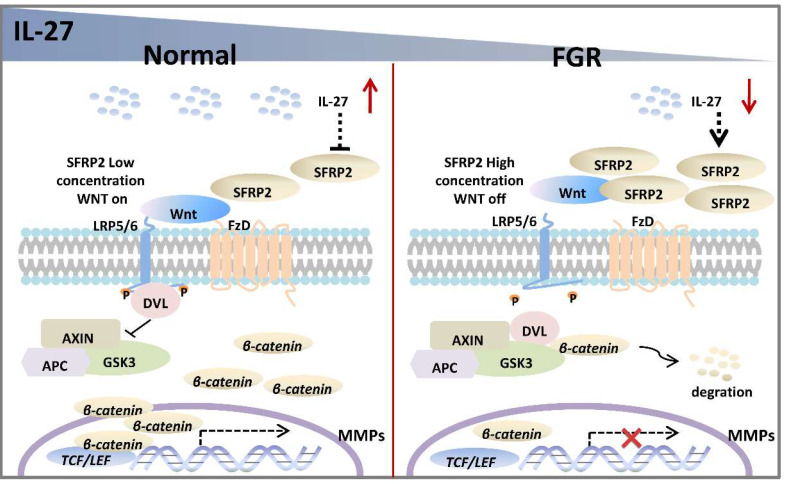
Potential roles of IL-27 in regulating SFRP2/Wnt/β-catenin pathway in normal pregnancy and FGR. In normal placentae, IL-27 is negatively associated with SFRP2, and thus activates Wnt/β-catenin signaling to turn on the expression of target genes (like MMPs), which may further facilitate the invasion and migration of trophoblast cells. However, in FGR placentae, the decreased IL-27 fails to maintain the activity of canonical Wnt pathway, which may impair bio-functions of trophoblasts and inhibit placental development.

In the current study, we assume that the IL-27/IL-27 signaling may decrease the expression of SFRP2, which will abrogate its inhibitory effect on Wnt/β‐catenin pathway and thus promote the transcription of genes associated with trophoblast proliferation, migration and invasion in normal pregnancy. However, FGR placentae presents an impaired IL-27/IL-27RA signaling, and SFRP2 is upregulated with inhibited Wnt/β‐catenin pathway, in which way abnormal trophoblast bio-functions may result in aberrant alterations of FGR placentae (**Figure 6**). The study of tentative therapeutic methods targeting IL-27/IL-27RA/Wnt or SFRP2 is of great research significance in investigating trophoblast-related diseases, such as FGR, preeclampsia, miscarriage and so on.

## Figures and Tables

**Table 1:** Gene primers for qRT-PCR

*ACTIN* (human)	Forward	CTACCTCATGAAGATCCTCACC
	Reverse	AGTTGAAGGTAGTTTCGTGGAT
*IL27* (human)	Forward	CCTGGTTCAAGCTGGTGTCT
	Reverse	AAGGGCTGAAGCGTGGTG
*IL27RA* (human)	Forward	CCCGTCTTCGTGAACCTA
	Reverse	AGCGGCCATACACTTTGT
*SFRP2* (human)	Forward	CCAAGAAGTTCCTGTGCTCG
	Reverse	GGATGCAAAGGTCGTTGTCC
*CCND1* (human)	Forward	CCTTCGTTGCCCTCTGTG
	Reverse	TCCTCCTCTTCCTCCTCC
*CMYC* (human)	Forward	CGACTCTGAGGAGGAACA
	Reverse	CTCTTGGCAGCAGGATAG
*SOX9* (human)	Forward	GCTCAAAGGCTACGACTG
	Reverse	GTGGTCCTTCTTGTGCTG
*MMP2* (human)	Forward	CCACTGCCTTCGATACAC
	Reverse	GAGCCACTCTCTGGAATCTTAAA
*MMP9* (human)	Forward	TTGACAGCGACAAGAAGTGG
	Reverse	GCCATTCACGTCGTCCTTAT
*Actin* (mouse)	Forward	CAGCCTTCCTTCTTGGGTAT
	Reverse	TGATCTTGATCTTCATGGTGC
*Sfrp2* (mouse)	Forward	CTAGTAGCGACCACCTCCTG
	Reverse	GCACGGATTTCTTCAGGTCC
*Ccnd1* (mouse)	Forward	TCCCTTGACTGCCGAGAA
	Reverse	GAGGGTGGGTTGGAAATG
*Cmyc* (mouse)	Forward	GTGGTCTTTCCCTACCCG
	Reverse	GCTGTGCGGAGGTTTGCT
*Sox9* (mouse)	Forward	CAGGTGCTGAAGGGCTAC
	Reverse	TCCTCCACGAAGGGTCTC

## Abbreviations

DEGs: differentially expressed genes
FGR: fetal growth restriction
GO: gene ontology
GSEA: gene set enrichment analysis
IL-27R: interleukin-27 receptor
IL-27RA: interleukin-27 receptor α
MMP-2/9: Matrix metallopeptidase 2/9
SFRP2: secreted frizzled-related protein 2
